# Supplementary material for: Comparative genomic analysis of Methylocystis sp. MJC1 as a platform strain for polyhydroxybutyrate biosynthesis
Source: PLoS One. 2023 May 10;18(5):e0284846. doi: 10.1371/journal.pone.0284846 (PMC10171618; doi:10.1371/journal.pone.0284846)
Supplement: S1 Table — AAI values are provided in percentage. Methylocystis sp. MJC1 had total 4306 genes identified during AAI. (DOCX) [file pone.0284846.s001.docx]

**Supplemental Table 1.** Average Amino acid Identity (AAI) comparison between *Methylocystis* sp. MJC1 and *Methylocystis* genus clade. AAI values are provided in percentage. *Methylocystis* sp. MJC1 had total 4306 genes identified during AAI.

| Strain name | Total number of genes | Number of orthologous genes shared with *Methylocystis* sp. MJC1 | Orthologous fraction (OF) shared with *Methylocystis* sp. MJC1 | Mean AAI | Std AAI |
| --- | --- | --- | --- | --- | --- |
| *Methylocystis parvus* OBBP | 4302 | 2765 | 64.27 | 80.49 | 12 |
| *Methylocystis parvus* (ASM968519v1) | 4338 | 2801 | 65.05 | 80.41 | 12.12 |
| *Methylocystis* sp. ATCC 49242 strain Rockwell | 4643 | 2527 | 58.69 | 78.64 | 12.71 |
| *Methylocystis* strain SB2 | 3521 | 2273 | 64.56 | 74.67 | 12.54 |
| *Methylocystis* sp. B8 | 3363 | 2178 | 64.76 | 74.62 | 12.81 |
| *Methylocystis rosea* | 4113 | 2325 | 56.53 | 74.54 | 12.86 |
| *Methylocystis* sp. FS | 3793 | 2273 | 59.93 | 74.52 | 12.57 |
| *Methylocystis* *hirsuta* | 4177 | 2365 | 56.62 | 74.5 | 12.84 |
| *Methylocystis rosea* | 3696 | 2286 | 61.85 | 74.5 | 12.82 |
| *Methylocystis* sp. H4A | 4289 | 2499 | 58.27 | 74.44 | 12.61 |
| *Methylocystis* sp. SC2 | 3621 | 2272 | 62.75 | 74.4 | 12.84 |
| *Methylocystis* sp. L43 | 3974 | 2308 | 58.08 | 74.4 | 12.82 |
| *Methylocystis* sp. MitZ-2018 | 4183 | 2347 | 56.11 | 74.37 | 12.96 |
| *Methylocystis* sp. H62 | 4481 | 2282 | 53 | 74.28 | 12.75 |
| *Methylocystis* sp. H15 | 3967 | 2272 | 57.27 | 74.23 | 12.76 |
| *Methylocystis heyeri* | 4261 | 2443 | 57.33 | 69.73 | 13.38 |
| *Methylocystis* sp. LW5 | 4379 | 2306 | 53.55 | 69.04 | 13.16 |
| *Methylocystis bryophila* | 4368 | 2225 | 51.67 | 68.16 | 13.41 |
