# Supplementary material for: Comparative genomic analysis of Methylocystis sp. MJC1 as a platform strain for polyhydroxybutyrate biosynthesis
Source: PLoS One. 2023 May 10;18(5):e0284846. doi: 10.1371/journal.pone.0284846 (PMC10171618; doi:10.1371/journal.pone.0284846)
Supplement: S2 Table — ANI values are provided in percentage. (DOCX) [file pone.0284846.s002.docx]

**Supplemental Table 2.** Average Nucleotide Identity (ANI) comparison between *Methylocystis* sp. MJC1 and *Methylocystis* genus clade. ANI values are provided in percentage.

| Strain name | ANI with *Methylocystis* sp. MJC1, % |
| --- | --- |
| *Methylocystis parvus* sp. BRCS2 | 85.4443591 |
| *Methylocystis parvus* OBBP | 85.4434873 |
| *Methylocystis* sp. ATCC 49242 | 85.2546069 |
| *Methylocystis rosea* sp. GW6 | 84.5988424 |
| *Methylocystis* sp. H4A | 84.4300687 |
| *Methylocystis* sp. SC2 | 84.388883 |
| *Methylocystis* sp. H62 | 84.3730907 |
| *Methylocystis* *rosea* sp. BRCS1 | 84.3081114 |
| *Methylocystis* sp. SB2 | 84.2859737 |
| *Methylocystis* sp. H15 | 84.2743489 |
| *Methylocystis* sp. L43 | 84.2734146 |
| *Methylocystis* sp. MitZ 2018 | 84.2703885 |
| *Methylocystis* sp. FS | 84.2611398 |
| *Methylocystis* *hirsuta* sp. CSC1 | 84.2598418 |
| *Methylocystis* *bryophila* sp. S285 | 84.1783224 |
| *Methylocystis* sp. B8 | 84.1741623 |
| *Methylocystis* *heyeri* sp. H2 | 84.1691712 |
| *Methylocystis* sp. LW5 | 84.1321801 |
