# Supplementary material for: Comparative genomic analysis of Methylocystis sp. MJC1 as a platform strain for polyhydroxybutyrate biosynthesis
Source: PLoS One. 2023 May 10;18(5):e0284846. doi: 10.1371/journal.pone.0284846 (PMC10171618; doi:10.1371/journal.pone.0284846)
Supplement: S3 Table — The names of major biochemical pathways or enzymes are written in bold. (DOCX) [file pone.0284846.s003.docx]

**Supplemental Table 3.** Central carbon metabolism-related genes in *Methylocystis* sp. MJC1. The names of major biochemical pathways or enzymes are written in bold.

| Locus tags in *Methylocystis* sp. MJC1 | Protein/function | Gene name |
| --- | --- | --- |
| **Particulate methane monooxygenase** | | |
| JMJ47_001059 | Particulate methane monooxygenase subunit C2 | *pmoC2* |
| JMJ47_001635 or JMJ47_001564 or JMJ47_000470 | Particulate methane monooxygenase subunit C1 | *pmoC1* |
| JMJ47_001636 or JMJ47_000471 | Particulate methane monooxygenase subunit A1 | *pmoA1* |
| JMJ47_001637 or JMJ47_000472 | Particulate methane monooxygenase subunit B1 | *pmoB1* |
| JMJ47_000185 | Particulate methane monooxygenase subunit C1 | *pmoC1* |
| JMJ47_002652 | Particulate methane monooxygenase subunit C2 | *pmoC2* |
| JMJ47_002653 | Particulate methane monooxygenase subunit A2 | *pmoA2* |
| JMJ47_002654 | Particulate methane monooxygenase subunit B2 | *pmoB2* |
| **Soluble methane monooxygenase** | | |
| JMJ47_001409 | Soluble methane monooxygenase component X | *mmoX* |
| JMJ47_001410 | Soluble methane monooxygenase component Y | *mmoY* |
| JMJ47_001412 | Soluble methane monooxygenase component Z | *mmoZ* |
| JMJ47_001411 | Soluble methane monooxygenase component B | *mmoB* |
| JMJ47_001414 | Soluble methane monooxygenase component C | *mmoC* |
| JMJ47_001413 | Soluble methane monooxygenase component D | *mmoD* |
| **Methanol dehydrogenase** | | |
| JMJ47_003571 | PQQ-dependent dehydrogenase | *mxaF* |
| JMJ47_003570 | Methanol oxidation system protein | *moxJ* |
| JMJ47_003569 | Cytochrome *c*-L | *mxaG* |
| JMJ47_003568 | Methanol dehydrogenase small beta subunit | *mxaI* |
| JMJ47_003567 | MoxR family ATPase | *moxR* |
| JMJ47_003566 | MxaS protein, involved in methanol oxidation | *mxaS* |
| JMJ47_003565 | MxaA protein involved in Ca^2+^ insertion into methanol dehydrogenase | *mxaA* |
| JMJ47_003564 | MxaC protein involved in Ca^2+^ insertion into methanol dehydrogenase | *mxaC* |
| JMJ47_003563 | MxaK protein involved in Ca^2+^ insertion into methanol dehydrogenase | *mxaK* |
| JMJ47_003562 | MxaL protein involved in Ca^2+^ insertion into methanol dehydrogenase | *mxaL* |
| JMJ47_003561 | MxaD-like protein | *mxaD* |
| JMJ47_003560 | Putative MxaH-like protein | *mxaH* |
| JMJ47_000170 | Putative methanol oxidation protein | *xoxJ* |
| JMJ47_000169 | C-type cytochrome methanol metabolism-related | *xoxG* |
| JMJ47_000168 | PQQ-dependent dehydrogenase | *xoxF* |
| **Pyrroloquinoline quinone (PQQ) biosynthesis** | |  |
| JMJ47_001596 | Pyrroloquinoline quinone biosynthesis protein B | *pqqB* |
| JMJ47_001595 | Pyrroloquinoline quinone biosynthesis protein C | *pqqC* |
| JMJ47_001594 | Pyrroloquinoline quinone biosynthesis protein E | *pqqE* |
| JMJ47_003557 | pyrroloquinoline quinone precursor peptide | *pqqA* |
| **Tetrahydromethanopterin pathway** | | |
| JMJ47_000916 | H_4_MPT-linked C_1_ transfer pathway protein | - |
| JMJ47_000175 or JMJ47_000915 | Formaldehyde-activating enzyme | *fae* |
| JMJ47_002643 | Formaldehyde-activating enzyme | *fae* |
| JMJ47_000174 | Formaldehyde-activating enzyme | *fae* |
| JMJ47_000914 | NAD(P)-dependent methylenetetrahydromethanopterin  dehydrogenase | *mtd* |
| JMJ47_000180 | Methenyltetrahydromethanopterin cyclohydrolase | *mch* |
| JMJ47_002462 | Formylmethanofuran-tetrahydromethanopterin  N-Formyltransferase | *ftr* |
| JMJ47_002463 | Formylmethanofuran dehydrogenase subunit A | *fwdA* |
| JMJ47_002464 | Formylmethanofuran dehydrogenase subunit B | *fwdB* |
| JMJ47_002461 | Formylmethanofuran dehydrogenase subunit C | *fwdC* |
| JMJ47_003510 |  | *fdwA* |
| JMJ47_000732 | NAD-dependent formate dehydrogenase,  alpha subunit | - |
| JMJ47_000731 | Formate dehydrogenase subunit beta | *fdwB* |
| JMJ47_000730 | Formate dehydrogenase subunit gamma | *fdsG* |
| JMJ47_000734 | NAD-dependent formate dehydrogenase  delta subunit | - |
| JMJ47_004028 | NAD-dependent formate dehydrogenase | *fdh* |
| JMJ47_000733 | Formate dehydrogenase family accessory protein | - |
| **H4Folate pathway** | | |
| JMJ47_000489 | formate-tetrahydrofolate ligase | *fhs* |
| JMJ47_000493 | methenyltetrahydrofolate cyclohydrolase | *folD* |
| JMJ47_000492 | methylenetetrahydrofolate dehydrogenase | *folD* |
| **Serine cycle** | | |
| JMJ47_002407 | serine hydroxymethyltransferase | *glyA* |
| JMJ47_003359 | serine hydroxymethyltransferase | *glyA* |
| JMJ47_000490 | serine-pyruvate aminotransferase | *agxt* |
| JMJ47_000491 | glycerate dehydrogenase | *hprA* |
| JMJ47_000130 | D-glycerate dehydrogenase | *hprA* |
| JMJ47_000498 | glycerate kinase | *gckA* |
| JMJ47_002940 | phosphopyruvate hydratase | *eno* |
| JMJ47_000496 | phosphoenolpyruvate carboxylase | *pck* |
| JMJ47_000407 | phosphoenolpyruvate carboxylase | *pck* |
| JMJ47_000910 | malate dehydrogenase | *mdh* |
| JMJ47_000494 | malate-CoA ligase | *mtkA* |
| JMJ47_000497 | malyl-CoA lyase | *mclA* |
| JMJ47_000081 | malyl-CoA lyase | *mclA* |
| **Ethylmalonyl-CoA pathway** | | |
| JMJ47_000580 or JMJ47_000749 | Acetyl-CoA C acetyltransferase | *atoB1* |
| JMJ47_000748 | Acetoacetyl-CoA reductase 1 | *phaB1* |
| JMJ47_003692 | R-3-hydroxybutyryl-CoA dehydratase | *croR* |
| JMJ47_000106 | Crotonyl-CoA reductase carboxylase | *ccr* |
| JMJ47_002995 | Ethylmalonyl-CoA epimerase | *epi* |
| JMJ47_000106 | Crotonyl-CoA reductase | *ccr* |
| JMJ47_002101 | Butyryl-CoA carboxylase | *-* |
| JMJ47_000104 | Ethylmalonyl-CoA mutase | *ecm* |
| JMJ47_000072 | Methylsuccinyl-CoA dehydrogenase | *msd* |
| JMJ47_002032 | Mesaconyl-CoA hydratase | *mch* |
| JMJ47_000497 | β-methylmalyl-CoA lyase | *mclA1* |
| JMJ47_002101 and JMJ47_003386 | Propionyl-CoA carboxylase 1 | *pcc* |
| JMJ47_002995 | methylmalonyl-CoA epimerase | *mcee* |
| JMJ47_003174 or JMJ47_003326 | Methylmalonyl-CoA mutase | *mmut* |
| JMJ47_003701 | 3-hydroxybutyryl-CoA dehydrogenase | *fadB2* |
| **PHB synthesis** | | |
| JMJ47_000824 | Class I poly-(R)-hydroxyalkanoic acid synthase | *phaC* |
| JMJ47_000748 | acetoacetyl-CoA reductase | *phbB* |
| JMJ47_000749 | acetoacetyl-CoA reductase | *phbA* |
| JMJ47_000750 | Polyhydroxyalkanoate synthesis repressor PhaR | *phaR* |
| JMJ47_002920 | Polyhydroxyalkanoate depolymerase | - |
| JMJ47_003936 | Poly(3-hydroxyalkanoate) synthetase | - |
| JMJ47_003319 | Phasin | - |
| JMJ47_001946 | Phasin family protein | - |
| **Tricarboxylic acid cycle** | | |
| JMJ47_000910 | malate dehydrogenase | *mdh* |
| JMJ47_000494 | succinate--CoA ligase subunit beta | *sucC* |
| JMJ47_000495 | succinate--CoA ligase subunit alpha | *sucD* |
| JMJ47_002844 | succinate--CoA ligase subunit beta | *sucC* |
| JMJ47_003217 | succinate--CoA ligase subunit alpha | *sucD* |
| JMJ47_002190 | citrate (Si)-synthase | *cs* |
| JMJ47_003772 | succinate dehydrogenase cytochrome b556 subunit | *sdh1* |
| JMJ47_003771 | succinate dehydrogenase hydrophobic membrane anchor protein | *sdh2* |
| JMJ47_001758 | succinate dehydrogenase flavoprotein subunit | *sdh3* |
| JMJ47_001759 | succinate dehydrogenase iron-sulfur subunit | *sdh4* |
| JMJ47_000007 | fumarate hydratase class II | *fh* |
| JMJ47_000043 | Aconitate hydratase A | *acnA* |
| JMJ47_003204 | isocitrate dehydrogenase | *idh* |
| JMJ47_003204 | isocitrate dehydrogenase | *idh* |
| JMJ47_001886 | 2-oxoglutarate dehydrogenase | *sucA* |
| JMJ47_003218 | 2-oxoglutarate dehydrogenase E1 component | *sucA* |
| JMJ47_003219 | dihydrolipoamide succinyltransferase, a 2-oxoglutarate system E2 component | *sucB* |
| JMJ47_002924 | dihydrolipoyl dehydrogenase, an E3 component of three alpha-ketoacid dehydrogenase complexes | *dld* |
| **RuMP pathway and glycolysis** | | |
| JMJ47_001021 | glucose-6-phosphate isomerase | *pgi* |
| JMJ47_002986 | ribose 5-phosphate isomerase A | *rpi* |
| JMJ47_002799 | Ribulose 5-phosphate 3-epimerase | *rpe* |
| JMJ47_002794 | transketolase | *tkt1* |
| JMJ47_002794 | transketolase | *tkt2* |
| JMJ47_001830 | phosphogluconate dehydratase | *edd* |
| JMJ47_003380 | pyrophosphate--fructose-6-phosphate 1-phosphotransferase | *pfp* |
| JMJ47_003380 | Phosphofructokinase (ppi-dependent)/Pyrophosphate-dependent Phosphofructokinase (s7p) | *pfp* |
| JMJ47_002796 | fructose-1-6-bisphosphate aldolase | *fbaA2* |
| JMJ47_001617 | triose-phosphate isomerase | *tpi* |
| JMJ47_000253 | type I glyceraldehyde-3-phosphate dehydrogenase | *gapdh* |
| JMJ47_000252 | phosphoglycerate kinase | *pgk* |
| JMJ47_002940 | phosphopyruvate hydratase | *eno* |
| JMJ47_001668 | pyruvate kinase | *pk* |
| **Nitrogen fixation** | | |
| JMJ47_002184 | Putative ferredoxin protein | *fixX* |
| JMJ47_002183 | Flavoprotein-ubiquinone oxidoreductase | *fixC* |
| JMJ47_002182 | Electron transfer flavoprotein, alpha subunit | *fixB* |
| JMJ47_002181 | Electron transfer flavoprotein beta-subunit | *fixA* |
| JMJ47_002180 | Nitrogenase-stabilizing/protective protein | *nifW* |
| JMJ47_002179 | NifP protein, serine O-acetyltransferase | *-* |
| JMJ47_002178 | NifV protein, encodes a homocitrate synthase | *nifV* |
| JMJ47_002175 | NifU-like iron-sulfur cluster assembly protein | *-* |
| JMJ47_002174 | Putative nitrogen fixation protein | *nifQ* |
| JMJ47_002156 | Nitrogen fixation protein | *fixU* |
| JMJ47_002177 | Nitrogenase metalloclusters biosynthesis protein | *nifS* |
| JMJ47_002176 | Putative NifU protein | *nifU* |
| JMJ47_002172 | NifX-associated protein | *-* |
| JMJ47_002171 | NifX-associated protein | *-* |
| JMJ47_002170 | Nitrogen fixation protein | *nifX* |
| JMJ47_002169 | Nitrogenase molybdenum-iron cofactor biosynthesis protein | *nifN* |
| JMJ47_002168 | Nitrogenase MoFe cofactor biosynthesis protein | *nifE* |
| JMJ47_002167 | Nitrogenase molybdenum-iron protein beta chain | *nifD* |
| JMJ47_002166 | Nitrogenase protein alpha chain | *nifD* |
| JMJ47_002165 | Nitrogenase iron protein, Nitrogenase reductase | *nifH* |
| JMJ47_002167 | B-subunit of Component 1 of nitrogenase | *nifK* |
| JMJ47_002154 | NifZ family protein | *-* |
| JMJ47_002153 | LRV FeS4 cluster domain protein | *-* |
| JMJ47_002173 | 4Fe-4S ferredoxin, nitrogenase-associated protein | *-* |
| JMJ47_002148 | Nif-specific regulatory protein | *nifA* |
| JMJ47_002153 | LRV FeS cluster domain protein | *-* |
| JMJ47_002151 | Putative iron-sulfur cluster assembly protein | *-* |
| JMJ47_002150 | Ferredoxin-like protein | *-* |
| JMJ47_002149 | Nitrogenase FeMo cofactor biosynthesis protein | *nifB* |
| **Nitrate/nitrite assimilation** | | |
| JMJ47_000756 | Nitrate ABC transporter permease | *nrtB* |
| JMJ47_000757 | Nitrate ABC transporter ATP-binding protein | *nrtC* |
| JMJ47_000758 | Nitrate ABC transporter substrate-binding protein | *nrtA* |
| JMJ47_000190 | Nitrate reductase | *-* |
| JMJ47_003888 | Nitrate transporter component | *nrtA* |
| JMJ47_003884 | Nitrite reductase [NAD(P)H], large subunit | *nasD* |
| JMJ47_003883 | NAD(P)H-dependent nitrite reductase catalytic subunit | *nirA* |
| JMJ47_003882 | NAD(P)H-dependent nitrite reductase flavoprotein subunit | *-* |
| **Ammonium assimilation** | | |
| JMJ47_001366 | Putative ammonium transporter | *-* |
| JMJ47_001558 | Putative ammonium transporter | *-* |
| JMJ47_002871 | Glutamine synthetase (GS) | *-* |
| JMJ47_001687 | Glutamate synthetase (GOGAT), large subunit | *-* |
| JMJ47_001688 | Glutamate synthetase (GOGAT), small subunit | *-* |
| JMJ47_003107 | Glutamate dehydrogenase (NAD)/Glutamate dehydrogenase (NADP) |  |
| **Nitrogen metabolism-related regulatory components** | | |
| JMJ47_000014 | RNA polymerase sigma factor RpoN | *rpoN* |
| JMJ47_001935 | tRNA-dihydrouridine synthase | *nifR3* |
| JMJ47_001934 | Signal transduction histidine kinase, nitrogen specific | *ntrB* |
| JMJ47_001933 | Nitrogen metabolism transcriptional regulator | *ntrC* |
| JMJ47_001928 | Multi-sensor signal transduction histidine kinase | *ntrY* |
| JMJ47_001927 | Nitrogen assimilation regulatory protein | *ntrX* |
| **Nitrification/ hydroxylamine detoxification** | | |
| JMJ47_002328 | Hydroxylamine oxidoreductase subunit A | *haoA* |
| JMJ47_002329 | Hydroxylamine oxidoreductase subunit B | *haoB* |
| JMJ47_003907 | Hydroxylamine reductase | *hcp* |
| **Denitrification** | | |
| JMJ47_001629 or JMJ47_002860 | Copper ABC transporter ATP-binding protein | *nosF* |
| JMJ47_003238 | ApbE family lipoprotein | *nosX* |
| JMJ47_002119 | NnrS protein (probable denitrification associated gene) | *-* |
| JMJ47_002992 | NnrU family protein (probable denitrification associated gene) | *-* |
