# Supplementary material for: Comparative genomic analysis of Methylocystis sp. MJC1 as a platform strain for polyhydroxybutyrate biosynthesis
Source: PLoS One. 2023 May 10;18(5):e0284846. doi: 10.1371/journal.pone.0284846 (PMC10171618; doi:10.1371/journal.pone.0284846)
Supplement: S4 Table — The names of super categories are written in bold uppercase letters. (DOCX) [file pone.0284846.s004.docx]

**Supplemental Table 4.** Cluster of orthologous genes (COG) functional classification for unique genes identified in *Methylocystis* sp. MJC1 by pangenome analysis. The names of super categories are written in bold uppercase letters.

| Category | Functional Classification | Number of genes |
| --- | --- | --- |
| **CELLULAR PROCESSES AND SIGNALING** | | |
| D | Cell cycle control, cell division, chromosome partitioning | 3 |
| M | Cell wall/membrane/envelope biogenesis | 32 |
| N | Cell motility | 17 |
| O | Post-translational modification, protein turnover and chaperones | 7 |
| T | Signal transduction mechanisms | 65 |
| U | Intracellular trafficking, secretion, and vesicular transport | 20 |
| V | Defense mechanisms | 12 |
| W | Extracellular structures | 0 |
| Y | Nuclear structure | 0 |
| Z | Cytoskeleton | 0 |
| **INFORMATION STORAGE AND PROCESSING** | | |
| A | RNA processing and modification | 0 |
| B | Chromatin structure and dynamics | 0 |
| J | Translation, ribosomal structure, and biogenesis | 1 |
| K | Transcription | 43 |
| L | Replication, recombination, and repair | 17 |
| **METABOLISM** | | |
| C | Energy production and conversion | 23 |
| E | Amino acid transport and metabolism | 11 |
| F | Nucleotide transport and metabolism | 2 |
| G | Carbohydrate transport and metabolism | 9 |
| H | Coenzyme transport and metabolism | 9 |
| I | Lipid transport and metabolism | 17 |
| P | Inorganic ion transport and metabolism | 16 |
| Q | Secondary metabolites biosynthesis, transport, and catabolism | 8 |
| **POORLY CHARACTERIZED** | | |
| R | General function prediction only | 36 |
| S | Function unknown | 45 |
