# Supplementary material for: Comparative genomic analysis of Methylocystis sp. MJC1 as a platform strain for polyhydroxybutyrate biosynthesis
Source: PLoS One. 2023 May 10;18(5):e0284846. doi: 10.1371/journal.pone.0284846 (PMC10171618; doi:10.1371/journal.pone.0284846)
Supplement: S1 Fig — The location of core genes for Methylocystis sp. MJC1 is highlighted in red box. MYA represents evolutionary age of genes in million years ago. (DOCX) [file pone.0284846.s006.docx]

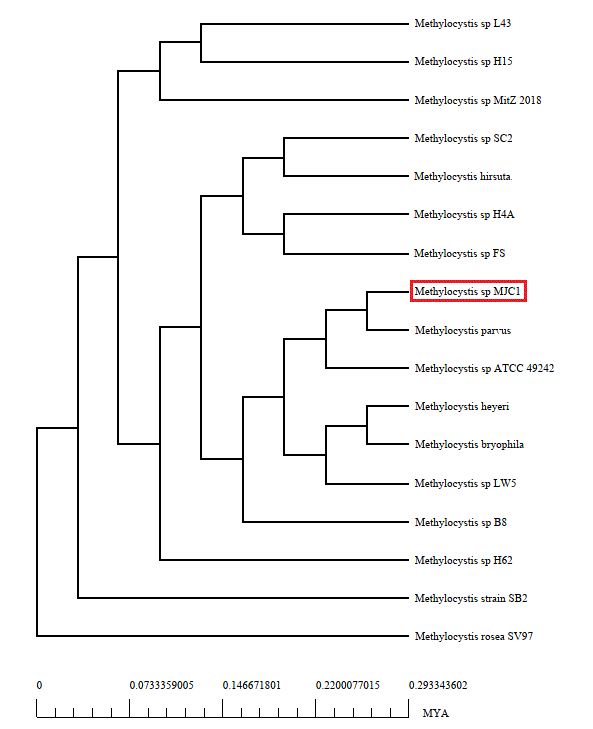


**Supplemental Figure S1.** Concatenated core gene (core genome) phylogenic tree for *Methylocystis* species strains. The location of core genes for *Methylocystis* sp. MJC1 is highlighted in red box. MYA represents evolutionary age of genes in million years ago.
